# Cytotoxicity and Mitochondrial Dysregulation Caused by α-Synuclein in *Dictyostelium discoideum*

**DOI:** 10.3390/cells9102289

**Published:** 2020-10-14

**Authors:** Sanjanie Fernando, Claire Y. Allan, Katelyn Mroczek, Xavier Pearce, Oana Sanislav, Paul R. Fisher, Sarah J. Annesley

**Affiliations:** Department of Physiology, Anatomy and Microbiology, La Trobe University, Bundoora 3086, Melbourne, Australia; sgfernando@students.latrobe.edu.au (S.F.); Claire.Allan@latrobe.edu.au (C.Y.A.); K.Mroczek@latrobe.edu.au (K.M.); xgpearce@students.latrobe.edu.au (X.P.); O.Sanislav@latrobe.edu.au (O.S.); p.fisher@latrobe.edu.au (P.R.F.)

**Keywords:** α-synuclein, mitochondria, AMPK, *Dictyostelium*

## Abstract

Alpha synuclein has been linked to both sporadic and familial forms of Parkinson’s disease (PD) and is the most abundant protein in Lewy bodies a hallmark of Parkinson’s disease. The function of this protein and the molecular mechanisms underlying its toxicity are still unclear, but many studies have suggested that the mechanism of α-synuclein toxicity involves alterations to mitochondrial function. Here we expressed human α-synuclein and two PD-causing α-synuclein mutant proteins (with a point mutation, A53T, and a C-terminal 20 amino acid truncation) in the eukaryotic model *Dictyostelium discoideum*. Mitochondrial disease has been well studied in *D. discoideum* and, unlike in mammals, mitochondrial dysfunction results in a clear set of defective phenotypes. These defective phenotypes are caused by the chronic hyperactivation of the cellular energy sensor, AMP-activated protein kinase (AMPK). Expression of α-synuclein wild type and mutant forms was toxic to the cells and mitochondrial function was dysregulated. Some but not all of the defective phenotypes could be rescued by down regulation of AMPK revealing both AMPK-dependent and -independent mechanisms. Importantly, we also show that the C-terminus of α-synuclein is required and sufficient for the localisation of the protein to the cell cortex in *D. discoideum*.

## 1. Introduction

Parkinson’s disease (PD) is the second most prevalent neurodegenerative disease and the most common movement disorder [1]. PD is characterized neuropathologically by the loss of dopaminergic neurons and the formation of Lewy bodies in the *substantia nigra* of the brain. Lewy bodies are intracytoplasmic inclusions composed mainly of aggregated α-synuclein as well as other proteins including α-synuclein-binding proteins, components of the ubiquitin-proteasome system, cytoskeletal proteins, proteins associated with signal transduction and phosphorylation and others [2]. Proteomic studies have identified more than 300 proteins in Lewy bodies [3], but α-synuclein remains the most abundant.

α-synuclein is a small (140 residues, 14.5kD), soluble, natively unfolded and heat stable, presynaptic protein which is encoded by the *SNCA* gene. It is expressed abundantly in the brain, especially in dopaminergic neurons [4]. *SNCA* was the first gene identified as being linked to an autosomal dominant form of familial PD and the mutation was an A53T substitution [5]. Mutations in *SNCA*, including duplications, triplications and point mutations are associated with autosomal, dominant, familial PD and form the basis for an increased risk of developing sporadic PD [6]. Several point mutations in the N-terminus have been identified, and the N-terminus is responsible for the aggregation properties of α-synuclein. In addition to the N-terminus, truncation of the C-terminus has been associated with PD [7]. Truncation of the C-terminus is associated with an increased propensity of α-synuclein to form fibrils, to aggregate faster than the full-length protein [8], and has been shown to increase toxicity of α-synuclein in fly and rodent models [8,9,10].

The localisation of α-synuclein is debatable. The protein was initially observed to be localised to the nuclear membrane and in presynaptic terminals of neurons [11]. Since then the protein has been reported to be present in multiple compartments including the nucleus, cytoplasm, plasma membrane and various cytoplasmic vesicles or exclusively localised to the cytoplasm of presynaptic terminals [12,13,14], to the cytosol and cell membranes [15,16], or to the mitochondria [17,18,19,20,21,22].

In addition to the mitochondrial localisation data, there are multiple lines of evidence that the accumulation of the protein α-synuclein and the selective loss of dopaminergic neurons are caused by defective mitochondrial energy metabolism, abnormal protein aggregation due to defective ubiquitin proteasomal system (UPS) and impairment of mechanisms protecting cells from oxidative stress and apoptosis [23,24,25].

The cellular energy-sensing protein AMP-activated protein kinase (AMPK) is activated by diverse cellular stresses, including oxidative stress and energy stress such as results from mitochondrial dysfunction. Its main role is to regulate energy (ATP) levels within the cell and it is regulated by the AMP:ATP ratio. In conditions of low ATP levels AMPK is activated and is able to phosphorylate many downstream targets acting broadly to inhibit energy consuming pathways and activate energy producing ones. The role of AMPK in PD has been studied in some detail and has been suggested to have neuroprotective effects. Metformin, an indirect AMPK activator (it inhibits complex I of the mitochondrial respiratory chain) [26,27], has been shown to reduce markers of inflammation, increase autophagic clearance of α-synuclein and decrease Reactive Oxygen Species in mice [28]. However, the extent to which these effects are due to activation of AMPK is unclear, since they were observed both in wild type and AMPK knockout mice [29]. Activation of AMPK has been shown to increase the clearance of α-synuclein from PD-like cells [28]. The exact mechanism of this clearance is unknown and evidence to date is contradictory [30]. Some studies indicate that α-synuclein promotes the phosphorylation and activation of AMPK, thereby stimulating downstream autophagic clearance of aggregates [31]. Others, however, suggest that α-synuclein reduces AMPK activation which would impair autophagy and exacerbate Lewy Body formation [32]. Further study is needed to characterise the roles of these key players in PD aetiology.

Studying complex human diseases in simple models can provide useful information about the underlying cellular pathology and provide mechanistic insights. Here we employ the simple eukaryotic model *Dictyostelium discoideum*. *D. discoideum* is a social amoeba or cellular slime mould that has long been recognized for its value as a biomedical model organism. The complete genome sequence of *D. discoideum* is known, it is genetically tractable, readily grown clonally as a eukaryotic microorganism and is highly accessible for biochemical, cell biological and physiological studies. These are properties it shares with other microbial model organisms. However, *D. discoideum* combines these with a unique life cycle, with motile unicellular and multicellular stages, and multiple cell types. This unique life cycle with unicellular and multicellular stages provides a variety of readily assayed, reproducible phenotypes which are “readouts” of the intracellular signalling pathways. This phenotypic richness, the fully sequenced mitochondrial [33] and nuclear [33,34] genomes and well-understood biochemistry and signalling pathways have made *D. discoideum* a fascinating model for studying cellular processes. The National Institute of Health (NIH) in the U.S.A. recognizes *D. discoideum* as a non-mammalian model organism for its particular value in biochemical research (http://www.nih.gov/science/models).

Mitochondrial disease has been created in *D. discoideum* by knocking down expression of essential nuclear-encoded mitochondrial proteins or by the disruption of several different mitochondrial genes (reviewed by Francione and Fisher [35]). In all cases, a clear set of defective phenotypes resulted. Among these were reduced growth on bacterial lawns and in liquid media, defective slug phototaxis and altered fruiting body morphology. Work by Bokko et al. [36] showed that these defective phenotypes were due to chronic activation of the cellular energy sensor kinase AMP-activated protein kinase (AMPK). Knocking down AMPK expression in mitochondrially diseased strains rescued the defective phenotypes, while expression of a constitutively active form of AMPK mimicked the mitochondrial disease phenotypes.

Because the cytopathological consequences of mitochondrial dysfunction are so well studied in *Dictyostelium discoideum,* it provides an ideal opportunity to determine if α-synuclein exerts evolutionarily conserved, dominant cytotoxic effects on mitochondria. The *Dictyostelium* genome does not encode a homologue of α-synuclein, so that the cytotoxicity of wild type and mutant forms of the protein can be studied easily in the absence of complications caused by the presence of the endogenous, native protein. To do this, we expressed three different forms of α-synuclein in the *D. discoideum* strain AX2, the full length α-synuclein referred to here as “wt α-synuclein”, an A53T point mutant and a C-terminally truncated α-synuclein lacking the last 20 amino acids of the wild type protein. We show here that in *Dictyostelium* the last 20 amino acids of α-synuclein are necessary and sufficient for the protein’s localisation to the cell cortex, that all three tested forms of α-synuclein are cytotoxic, but that the resulting phenotypes and their ability to be rescued by knock down of AMPK do not match the characteristic patterns seen in mitochondrially diseased strains. Consistent with this, direct respirometric assay of mitochondrial activities revealed that none of the mutant α-synuclein forms impair mitochondrial function.

## 2. Materials and Methods

### 2.1. Dictyostelium Strains, Culture Conditions and Development

*Dictyostelium* wild type strain, AX2, and the derived transformants were either grown axenically in HL-5 medium with glucose (Formedium, Hunstanton, Norfolk, UK), which for transformed cell lines contained 20 µg mL^−1^ G418 (Thermo Fisher, Waltham, MA, USA) in shaken suspension (150 rpm) at 21 °C or on SM agar plates (Formedium, Hunstanton, Norfolk, UK) with 20 µg mL^−1^ G418 and *Enterobacter aerogenes* as a food source. The transformants expressed either wild type α-synuclein (containing construct pPROF629), α-synuclein with an A53T point mutation (containing construct pPROF630), α-synuclein with a C-terminal truncation (containing construct pPROF631) or the 20 a.a. C-terminus of α-synuclein fused to GFP (containing construct pPROF632). Cotransformants were created which expressed one of the α-synuclein genes in combination with an antisense RNA (encoded by construct pPROF362) which inhibits expression of the catalytic alpha subunit of AMPK [36]. To compare the fruiting body morphologies of the wild type and mutant strains they were streak-diluted on SM agar plates containing a lawn of *E. aerogenes* supplemented with 20 µg mL^−1^ G418 for the transformant and cotransformant strains and grown at 21 °C until fruiting bodies were formed. The fruiting body morphology was observed using an Olympus SZ61TM dissecting microscope and photographs were taken by an attached Moticam 2300TM camera. In addition to the top view of fruiting bodies, agar slices were cut and flipped over to observe the side view.

### 2.2. Plasmid Construction

The human α-synuclein gene was amplified by PCR using a commercial cDNA clone (Origene^TM^, Rockville, MD, USA) containing the human *SNCA* gene (NM_000345.2) in the pCMV6-XL5 vector, as the template and primers SynprimF

(PGCGC **TCTAGA ATCGAT** ATG GATGTATTCATGAAAGGACTTTCAAAGGCC) and SynprimR

(PGCGC **TCTAGA CTCGAG** TTA GGCTTCAAGGTTCGTAGTCTTGATAC).

Two mutations of α-synuclein were also made, one with a G209A nucleotide substitution which produces an A53T amino acid replacement, and one with a C-terminal truncation removing the C-terminal 20 amino acids. The primers used to create the point mutant were Syn53mutprimF

(GAGTGGTGCATGGTGTG**A**CAACAGTGGCTGAG) and Syn53mutprimR

(CTCAGCCACTGTTG**T**CACACCATGCACCACTC). To create the truncated gene, the primers used were SynprimF and Syn120mutprimR

(PGCGC **TCTAGA CTCGAG** TTA AGGATCCACAGGCATATCTTCCAGAATTCC).

The PCR products were cloned into the *Xba*I (Promega, Madison, WI, USA) site of an *E. coli* vector pUC18. All three alleles were subcloned into the *Dictyostelium* vector pA15GFP using the restriction enzymes *Xho*I (Promega, Madison, WI, USA) and *Cla*I (Promega, Madison, WI, USA) replacing the GFP gene and named pPROF629, pPROF630 and pPROF631 expressing respectively the wild type, A53T mutant and C-terminally truncated forms of α-synuclein. For AMPK antisense inhibition the construct pPROF362 was used as described previously [36]. The C-terminal 60 nucleotides of the α-synuclein coding sequence were fused to the C-terminus of the AcGFP gene via PCR using the vector pAcGFP-C1(Clonetech^TM^, Rockville, MD, USA) as the template and the primers AcGFPSynCF

(GCGC**TCTAGAATCGAT**ATGGTGAGCAAGGGCGCCGAGCTG) and AcGFPSynCR (GCGC**TCTAGACTCGAG**TTAGGCTTCAGGTTCGTAGTCTTGATACCCTTCCTCAGAAGGCATTTCATAAGCCTCATTGTC CTTGTACAGCTCATCCATGCCGTGG).

The PCR amplicon was cloned into pA15GFP [37] using the restriction enzymes *Cla*I and *Xho*I, replacing the GFP gene. The resulting construct was named pPROF662.

### 2.3. Transformation

AX2 cells were transformed with 20 µg of construct DNA using the calcium phosphate DNA coprecipitation method [38]. Following selection on *Micrococcus luteus* lawns grown on SM agar plates containing 20 µg mL^−1^ of G418 [39], purified transformants were subcultured and maintained on *E. aerogenes* lawns and axenically in HL-5.

### 2.4. Calculation of Construct Copy Numbers

#### 2.4.1. Quantitative Southern Blotting

Quantitative Southern Blotting was used to calculate the copy number of the α-synuclein-expressing strains. Purified plasmid DNA containing the DNA of interest was digested with *Xho*I and *Cla*I to release the α-synuclein DNA and a series of 2-fold dilutions was prepared starting with a concentration of approximately 20 mg mL^−1^. This series was used as a standard to assess construct copy number. The plasmid DNA was also digested with *Sca*I (Promega, Madison, WI, USA) to linearise the DNA and this series was used as a standard to assess DNA loading. Genomic DNA was extracted from the test strains using DNAzol^TM^ (Thermo Fisher Scientific, Waltham, MA, USA) according to the manufacturer’s instructions and an aliquot of each preparation was digested with *Xho*I and *Cla*I and another aliquot was digested with *Sca*I. The standard and the genomic DNA was run on a 1% agarose (Thermo Fisher, Waltham, MA, USA) gel. The lanes containing the construct copy number standards and X*ho*I/*Cla*I digested genomic DNA was transferred to a nylon membrane (Hybond N+, Cytiva, Marlborough, MA, USA) via Southern hybridisation followed by detection using an ECF labelled probe. The probe was constructed by PCR using the primers SynPrimF and SynPrimR and the Fluorescein-High Prime labelling mixture (Sigma-Aldrich, St. Louis, MO, USA) according to the manufacturer’s instructions and the gel was visualised using the Storm 860™ Fluoroimager (Cytiva, Marlborough, MA, USA). The gel containing standard and genomic DNA digested with *Sca*I was stained with Vistra Green solution according to the manufacturer’s instructions (Cytiva, Marlborough, MA, USA). At the completion of staining, the DNA was visualised using the Storm 860™ Fluoroimager (Cytiva, Marlborough, MA, USA) in blue fluorescence mode. The two generated images were analysed using the ImageQuant software. The fluorescence was measured for each band using the software and two standard curves were plotted. The quantity of hybridised and total loaded DNA of the test samples were calculated using these calibration curves and used to determine construct copy number.

#### 2.4.2. Quantitative PCR

The number of copies of AMPK in the cotransformants was determined by quantitative PCR using the primers AMPKantiRT-For (TGGTGGTCTATATGGCAGTG) and AMPKantiRT-Rev (CACCACCGGTTACATACTCC) and iQ SYBR Green Supermix according to the manufacturer’s instructions (Bio-Rad, Hercules, CA, USA). Genomic AX2 DNA was used to create a standard curve for estimation of the quantity of gDNA, while standard curves for the purified plasmid construct DNA were used for determining the quantity of the inserted plasmid construct. The single copy gene for filamin was amplified as a loading control using the primers FIL1588F (CCCTCAATGATGAAGCC) and FIL1688R (CCATCTAAACCTGGACC). The reaction was performed using an iCycler IQ Multicolor Real-Time PCR Detection System (Bio-Rad, Hercules, CA, USA). The calculations of copy numbers for each construct were based on the quantities of the construct and genomic DNA present in each strain and the sizes (in base pairs) of the construct and whole genome. This yielded raw copy numbers which were then normalized against the copy number for the control parental strain.

### 2.5. Western Blotting

For Western blotting, 1 × 10^7^ cells were lysed in 200 mL of lysis buffer (50 mM Tris-HCl, 150 mM NaCl and 0.02% Triton-X100) centrifuged and the supernatant was transferred to a new tube. A 15 mL aliquot of protein extract in 4× loading buffer (62.5 mM, Tris-HCl, pH 6.8, 25% glycerol, 2% SDS, 0.01% bromophenol blue) was boiled for 5 min, subjected to 10% SDS-PAGE, transferred to PVDF membrane (Bio-Rad, Hercules, CA, USA) and incubated in rabbit monoclonal anti-alpha + beta synuclein antibody [EP1646Y] (Abcam, Cambridge, UK ab51252) (1:1000), antibody followed by incubation in secondary antibody (goat) anti-rabbit IgG Cross-Absorbed Secondary antibody AP (ThermoFisher, Waltham, MA, USA G-21079) 1:1000. Proteins were visualised using the STORM image analyser.

### 2.6. Growth in Axenic Medium

Growth rates in liquid medium were measured as previously described [36]. Exponentially growing axenic strains of wild type and transformant *D. discoideum* cell lines were inoculated into 50 mL of HL-5 medium to a final density of 1 × 10^4^ cells mL^−1^ in 150 mL flasks. The cultures were grown at 21 °C on an orbital shaker (Ratek, Boronia, VIC., Australia) at a speed of 150 rpm. Cell counts were taken twice daily using a haemocytometer (Bright Line, 0.1 mm deep) for a total of 100 h. The generation times were calculated by log-linear regression during the exponential phase of growth, using the R software environment for statistical computing and graphics [40].

### 2.7. Growth on Bacterial Lawns

As previously described [36], a scraping of amoebae was subcultured to the centre of each of two normal agar plates containing a lawn of *E. coli* B2. The plates were incubated at 21 °C and the diameter of the growing plaque was measured in mm at approximately 8 h intervals for a period of 100 h. The results were analysed by linear regression in R to calculate the plaque expansion rate for each strain.

### 2.8. Phagocytosis Assay

Bacterial uptake by *D. discoideum* strains (wild type and transformants) was determined by analysing consumption of an *E. coli* strain expressing the fluorescent protein DsRed [41] as previously described [36]. 5 × 10^6^ *D. discoideum* vegetative cells were harvested, washed and resuspended in 1 mL of 20 mM phosphate buffer and starved for 30 min with shaking (150 rpm) at 21 °C. One mL of the *E. coli* DSRed suspension was added to the cells and incubated for 30 min. 450 µL of cells were taken in duplicate at T = 0 min and T = 30 min. The amoebae were washed free of undigested bacteria by differential centrifugation in the presence of 5 mM sodium azide (Sigma-Aldrich, St. Louis, MI, USA) and their fluorescence was measured in a Modulus Fluorometer (Turner Biosystems, Sunnyvale, CA, USA) using a Special Module (530 nm excitation and 580 nm emission). The hourly rate of consumption of bacteria by a single amoeba was calculated from the increase in fluorescence over 30 min, the fluorescence signal per million bacteria (separately determined) and the amoebal density.

### 2.9. Pinocytosis Assay

Pinocytosis assays [42] were performed using fluorescein isothiocyanate (FITC)-dextran (Sigma-Aldrich, St. Louis, MI, USA, average mol. mass 70 kDa) as previously described [36]. 5 × 10^6^ *D. discoideum* vegetative cells were harvested, and resuspended in HL-5 containing 20 mg mL^−1^ FITC-dextran for 70 min. Duplicate aliquots were taken at T = 0 min and T = 70 min, the cells harvested, washed twice in 20 mM phosphate buffer and lysed by the addition of 2 mL of 0.25% (*v*/*v*) Triton X-100 in 100 mM Na_2_HPO_4_, pH 9.2. The fluorescence of the lysate was measured using the Modulus Fluorometer using the Green Module (525 nm excitation and 580–640 nm emission). The hourly rate of uptake of medium was calculated from the cell density, the increase in fluorescence over 70 min, and a separate calibration curve relating the fluorescence signal to the volume of fluorescent medium.

### 2.10. Phototaxis and Thermotaxis Assays

Qualitative phototaxis tests were performed as described [43] by transferring toothpick scrapings of amoebae from colonies growing on *E. aerogenes* lawns to the centres of charcoal agar plates (5% activated charcoal (Sigma-Aldrich, St. Louis, MI, USA), 1.0% agar technical (Thermo Fisher, Waltham, MA, USA). Phototaxis was scored after a 48 h incubation at 21 °C with a lateral light source.

Quantitative phototaxis [43] tests involved the harvesting of amoebae from mass plates, thoroughly washing them free of bacteria by differential centrifugation, suspending them in saline at the appropriate dilutions and inoculating 20 µL onto a 1 cm^2^ area in the centre of each charcoal agar plate. The resulting cell densities ranged from 1.5 × 10^6^ to 3.7 × 10^7^ cells/cm^2^. Phototaxis was measured after 48 h incubation at 21 °C with a lateral light source.

Quantitative thermotaxis assays used washed amoebae prepared as for quantitative phototaxis and plated at a density of 3 × 10^6^ amoebae/cm^2^. A 20 µL aliquot of cells at this dilution were plated onto a 1 cm^2^ area in the centre of water agar plates (1.0% agar) and incubated for 72 h in darkness on a heat bar producing a 0.2 °C/cm gradient at the agar surface. The arbitrary temperature units correspond to agar surface temperatures of 14 °C at T1 and increasing in 2 °C increments to 28 °C at T8, as measured at the centre of plates in separate calibration experiments. Slug trails were transferred to PVC discs, stained with Coomassie Blue (Sigma-Aldrich, St. Louis, MI, USA) and digitized. The orientation of the slug migration was analysed using directional statistics [44].

### 2.11. Immunofluorescence

*D. discoideum* strains were grown in HL-5 medium, 1 × 10^6^ cells were attached to sterile coverslips in six-well Costar^TM^ plates (Thermo Fisher, Waltham, MA, USA) at 21 °C for 1 hr. HL-5 was replaced with low-fluorescence medium (3.85 g/L glucose, 1.78 g/L proteose peptone, 0.45 g/L yeast extract, 0.485 g/L KH_2_PO_4_ and 1.2 g/L Na_2_HPO_4_∙12H_2_O; filter sterile) at 21 °C for 30 min. Coverslips were washed 2 × 2 min with PBS containing 0.05% bovine serum albumin (type V; Sigma-Aldrich, St. Louis, MI, USA), (BSA) and cells fixed with PBS containing 4% paraformaldehyde for 10 min at RT. Coverslips were washed 2 × 5 min in 20 mM NH_4_Cl in PBS and 2 × 5 min in PBS before permeabilisation with prechilled (−20 °C) methanol for 2 min, and washed again 2 × 5 min with PBS (0.05% BSA). Blocking was performed overnight at 4 °C in PBS containing 0.05% Tween 20, 1% BSA, 1% cold-water fish-skin gelatin (Sigma-Aldrich, St. Louis, MI, USA), and 0.02% sodium azide. Transformants expressing wildtype α-synuclein, α-synuclein with an A53T point mutation or α-synuclein with a C-terminal truncation were incubated for 4 h at RT with rabbit monoclonal anti-alpha + beta synuclein antibody [EP1646Y] 1:100 in blocking buffer. Transformants expressing the C-terminus of α-synuclein fused to GFP were incubated with rabbit anti-GFP polyclonal antibody (Thermo Fisher, Waltham, MA, USA, A-11122) 1:500 in blocking buffer. Coverslips were washed 4 × 5 min with PBS (0.05% BSA) and incubated for 1 h at RT with secondary antibody (goat) anti-rabbit Alexa-Fluor^™^488 conjugated IgG antibody (Thermo Fisher, Waltham, MA, USA, A11034) 1:100 in PBS (0.05% BSA), before washing 2 × 5 min with PBS and then once in milliQ H_2_0. Coverslips were mounted on slides using ProLong Gold antifade mountant with DAPI (Thermo Fisher, Waltham, MA, USA). Slides were viewed under 100× objective on an Olympus (Shinjuku City, Tokyo, Japan) BX61 fluorescent microscope and images captured with an Olympus DP80 camera.

### 2.12. Seahorse Respirometry

Mitochondrial respiratory function was measured as described [45] in *Dictyostelium* parental and α-synuclein-expressing strains using the Seahorse Extracellular Flux Analyser (Seahorse Biosciences, North Billerica, MA, USA) in combination with a series of drugs added in a sequential order (10 μM DCCD (N,N′-dicyclohexylcarbodimide, an ATP synthase inhibitor (Sigma-Aldrich, St. Louis, MI, USA), 10 μM CCCP (carbonyl cyanide 3-chlorophenol hydrazone, a protonophore (Sigma-Aldrich, St. Louis, MI, USA)), 20 μM rotenone (Com plex I inhibitor (Sigma-Aldrich, St. Louis, MI, USA)) and either 10 μM antimycin A (Complex III inhibitor (Sigma-Aldrich, St. Louis, MI, USA)) or 1.5 mM BHAM (benzohydroxamic acid, alternative oxidase (AOX) inhibitor (Sigma-Aldrich, St. Louis, MI, USA)). The Seahorse Extracellular Flux Analyser measures the Oxygen Consumption Rate (OCR) as a readout of mitochondrial respiration. Briefly, *Dictyostelium* amoebae were harvested, washed and resuspended in SIH assay medium (Formedium, Hunstanton, Norfolk, United Kingdom) supplemented with 20 mM sodium pyruvate and 5 mM sodium malate (pH 7.4). For each strain to be tested, 1 × 10^5^ cells were inoculated into each of eight Matrigel-coated wells in a 24-well assay plate for the Seahorse XFe24 Flux Analyser (Seahorse Biosciences, North Billerica, MA, USA) and allowed to attach for 30 min. Three measurement cycles, consisting of a 3 min mixing, 2 min wait and 3 min measurement time, were performed before any addition of drugs and after each sequential addition of the pharmacological agents. Each condition tested had eight replicate wells except for the antimycin and BHAM treatment which used 4 replicate wells. The parental AX2 strain was included in every experiment in four wells (two for each of the final antimycin A and BHAM injections). From the measurements before and after each pharmacological addition, averages of the basal OCR, maximum CCCP-uncoupled respiration rates, OCR devoted to ATP synthesis and OCR used by non-mitochondrial sources and attributed to proton leak were calculated.

## 3. Results

### 3.1. Human α-Synuclein Can Be Expressed in D. discoideum

**Figure 1:**
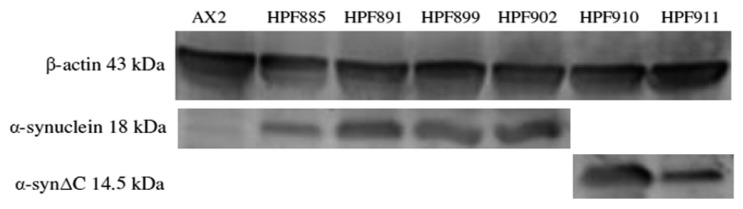
Human α-synuclein can be expressed in *D. discoideum.* Western blot showing the expression of α-synuclein WT HPF885 (38) and HPF891(96), α-synuclein A53T HPF899 (88) and HPF902(26) which runs at 18 kDa and α-synucleinΔC HPF910 (57) and HPF911(53) which runs at 14.5 kDa. The numbers in brackets represent construct copy numbers. Β-actin was detected as an indicator of sample load. The parental strain AX2 was run as a negative control.

The full length α-synuclein and α-synuclein with a A53T mutation or a C-terminal 20 amino acid deletion were cloned into a *Dictyostelium* expression vector (pA15GFP) and transformed into the wild type *D. discoideum* strain AX2. The constructs insert randomly into the genome through recombination and undergo rolling circle replication to generate strains, each of which contain different numbers of the constructs and hence different levels of expression [46]. Quantitative Southern blotting was used to determine the construct copy numbers and expression of α-synuclein was verified by Western blotting (Figure 1).

### 3.2. α-Synuclein Localizes to the Cell Cortex in D. discoideum and the C-Terminus Is Necessary and Sufficient for This to Occur

**Figure 2:**
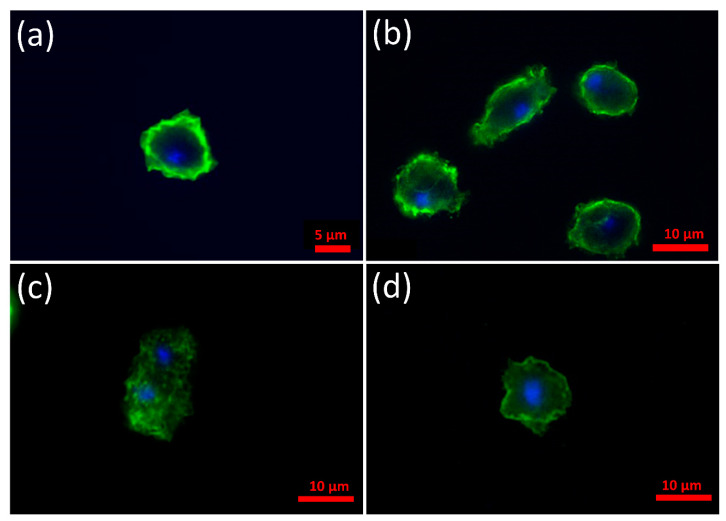
In *Dictyostelium,* α-synuclein concentrates in the cell cortex and the C-terminus is necessary and sufficient for this. Immunofluorescent images of strains expressing various forms of α-synuclein. The α-synuclein was detected using an anti-α-synuclein antibody and visualized with an Alexa-Fluor-488-conjugated secondary antibody. DAPI was used to detect the nucleus. (**a**) WT α-synuclein expressed in the parental strain AX2 (strain HPF891) is enriched in the cortex of the cell. (**b**) Strain HPF899 expressing α-synuclein with the point mutation A53T also exhibited enrichment at the cortex, indicating that the subcellular localization is not affected by this mutation. (**c**) Strain HPF912 expressing the C terminal truncated mutant α-synuclein. The mutated protein appears granulated throughout the cytoplasm with no cortical signal observed. This indicates that the 20 residues of the C-terminal are necessary for enrichment of the protein in the cell cortex. (**d**) Cells expressing the C-terminal 20 amino acids of α-synuclein fused to GFP and visualized using an anti-GFP antibody. A strong signal is obvious at the cell cortex which suggests that these amino acids are sufficient for localization of α-synuclein to the cell cortex in *Dictyostelium*.

Immunofluorescence microscopy was used to determine the localization of α-synuclein WT and the two mutant forms of α-synuclein in *D. discoideum*. Alpha-synuclein was detected using an anti-α-synuclein antibody together with an Alexa-Fluor-488-conjugated secondary antibody. The WT α-synuclein and the A53T point mutant were enriched at the cortex (Figure 2a,b respectively), whereas the C terminal truncation mutant was not enriched at the cortex and was present in the cytoplasm (Figure 2c). This showed that the C-terminal 20 residues are necessary for enrichment of the protein in the cell cortex. To investigate if the C-terminus was also sufficient for this localisation, the sequence encoding the C-terminal 20 amino acids were fused to a GFP reporter gene and the expression construct was transformed into the parental strain AX2. These strains expressing just the C-terminal 20 amino acids of α-synuclein which was visualized using an anti-GFP antibody and showed a strong signal at the cortex, suggesting that these amino acids are sufficient for localisation to the cortex in *Dictyostelium* (Figure 2d). The C-terminal region is known to be important for protein–protein interactions and could be facilitating such an interaction with proteins in the cortical regions near the plasma membrane. We also looked for mitochondrial colocalization using the dye Mitotracker Red and no colocalization of any of the three forms of α-synuclein with mitochondria was observed (Appendix A).

### 3.3. Mutant α-Synuclein Negatively Affects Phototaxis and Thermotaxis

In *Dictyostelium*, mitochondrial disease has been created genetically by the modification of several different mitochondrial genes [35,36,47,48] and in all cases a characteristic set of defective phenotypes resulted. These were reduced growth on bacterial lawns and in liquid medium, defective slug phototaxis and thermotaxis, altered fruiting body morphology with larger thicker stalks and unaltered endocytosis. These phenotypes were found to be due to the chronic activation of the cellular energy alarm protein AMP-activated protein kinase [36] (AMPK). To determine if expression of α-synuclein in *Dictyostelium* resulted in altered mitochondrial function, the characteristic set of mitochondrial disease phenotypes were investigated, beginning with phototaxis and thermotaxis.

**Figure 3:**
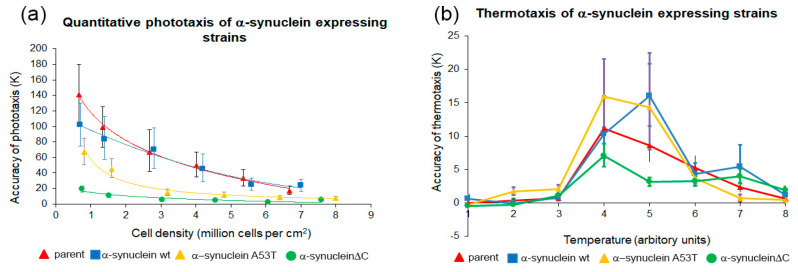
Mutant α-synuclein negatively affects phototaxis and thermotaxis. (**a**) Phototaxis. The parental strain (AX2), α-synuclein WT strain (HPF885), α-synuclein A53T mutant (HPF902) and α-synucleinΔC mutant (HPF905) were statistically analysed and the accuracy of phototaxis (κ) was plotted against their cell densities. When compared to AX2, the two mutant forms showed significantly decreased accuracy of phototaxis with the α-synucleinΔC mutant showing the lowest accuracy of phototaxis among the two forms. The α-synuclein WT strains showed a similar accuracy of phototaxis to AX2 at all cell densities. Error bars represent 90% confidence intervals. Lines were fitted with the least squares method to a logarithmic equation. (**b**) The accuracy of thermotaxis (κ) by slugs of the parental strain (AX2), and strains expressing the α-synuclein WT (HPF885), A53T point mutant (HPF902) and α-synucleinΔC mutant (HPF905) proteins was plotted against the temperature. The temperatures are expressed in arbitrary units from 1 to 8, shown in separate calibration experiments to correspond to agar surface temperatures of 14 °C to 28 °C at the centre of the plate [44]. When compared to AX2, strains expressing the α-synucleinΔC mutants show significantly reduced accuracy of thermotaxis while those expressing the α-synuclein WT or the α-synuclein A53T mutant form show a similar thermotaxis phenotype to AX2. Error bars represent 90% confidence intervals.

*Dictyostelium* slugs are capable of orientation towards light and in temperature gradients and this behaviour aids them in their migration to the surface of the soil, where optimal conditions for fruiting body formation and spore dispersal exist. Phototaxis is a phenotype that is very sensitive to alterations in energy levels and the accuracy of phototaxis is negatively affected in mitochondrial disease strains [35]. Phototaxis experiments were performed with the α-synuclein-expressing strains. Strains expressing wild type α-synuclein resembled the wild type AX2. Strains expressing A53T-α-synuclein displayed slight impairment of phototaxis, while the C-terminally truncated α-synuclein strains displayed a greater impairment of phototaxis compared to AX2 (Figure 3a). In *D. discoideum* the phototactic and thermotactic signalling pathways converge early and therefore share many downstream proteins [49]. Accordingly, most phototaxis genes are also essential for normal thermotaxis and the great majority of mutants with impaired phototaxis also exhibit defects in thermotaxis. The strains expressing wild type α-synuclein or A53T α-synuclein displayed accuracies of thermotaxis resembling the wild type AX2. The C-terminally truncated α-synuclein strains however displayed deranged thermotaxis with lower accuracies of thermotaxis at all temperature points compared to AX2. In contrast, the mild phototactic defect observed in the A53T α-synuclein strains was not accompanied by a significant thermotaxis defect (Figure 3b). This may be because the accuracies of thermotaxis were much smaller relative to measurement error, than phototaxis and hence small differences are harder to detect. The defects in phototaxis and thermotaxis observed in strains expressing the various forms of α-synuclein would be consistent with differing levels of severity of mitochondrial dysfunction, depending on the level of expression of the protein and the specific form being expressed. The results suggest that the wild type protein is the least toxic and the truncated protein the most toxic of the three forms we tested. This is in accord with findings in mammalian cells [50,51,52] and in PD mouse [52,53,54] and fly [10] models.

### 3.4. Strains Expressing C-Terminally Truncated α-Synuclein Display Aberrant Fruiting Bodies

**Figure 4:**
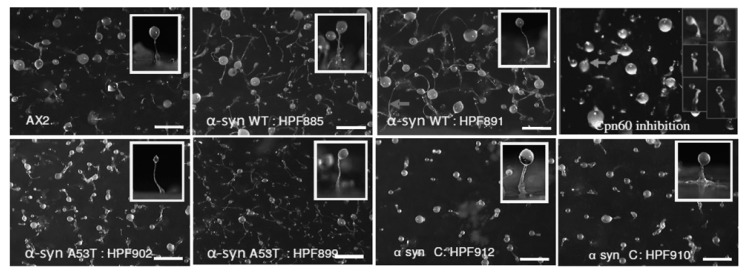
Multicellular morphogenesis of α-synuclein-expressing strains. Fruiting body morphology by the parental strain (AX2), strains expressing α-synuclein wild type (α-syn WT), point mutant (α-syn A53T), truncated (α-synΔC) α-synuclein and mitochondrial disease strain (Cpn60 inhibition). The α-syn WT and α-syn A53T produce thinner, longer stalks while the α-synΔC display fewer fruiting bodies with shorter and thicker stalks. Insets show the side view of a single fruiting body. The scale bar shows 1 mm.

When induced by starvation, the unicellular *Dictyostelium* can develop into a multicellular fruiting body through chemotactic aggregation. Mitochondrial disease in *Dictyostelium* results in fruiting bodies which have shorter, yet thicker stalks. The morphogenesis of all three classes of α-synuclein-expressing strains was observed and compared with the parental strain AX2 and a mitochondrial disease strain (Cpn60 inhibition). Figure 4 shows representative images of the fruiting body morphology of α-synuclein-expressing strains. The α-synuclein wild type and point mutant strains produced thin, long stalks like the parental strain, while strains expressing the C-terminally truncated form produced sparse fruiting bodies which had shorter and thicker stalks reminiscent of mitochondrial disease strains. This result is consistent with reports that the truncated α-synuclein is more toxic to mammalian cells than the A53T point mutant or wild type forms of the protein [8,50,51,52].

### 3.5. α-Synuclein Impairs Growth on Plates but Not in Liquid Medium

**Figure 5:**
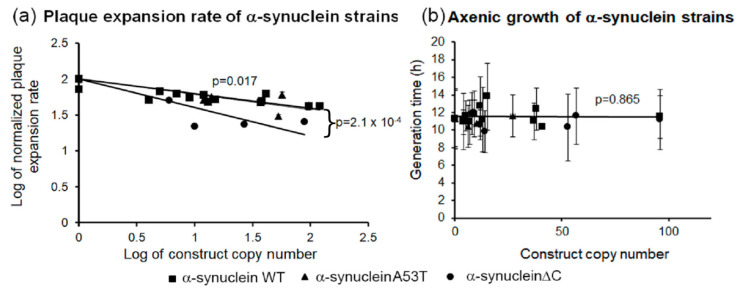
α-synuclein affects growth on bacterial lawns but not in liquid media. (**a**) The plaque expansion rates by the α-synuclein-expressing strains plotted against the α-synuclein construct copy number. The tested strains were grown on *E. coli* B2 lawns on SM agar and the plaque expansion was measured over one hundred hours. Experiments were performed in triplicate in three individual experiments. Alpha-synuclein WT and the point mutant strains showed slower growth compared to the parental strain but were not significantly different from each other, the α-synucleinΔC strains showed an even slower growth compared to the parental strain. Error bars represent standard errors of the mean. The regression was significant at indicated *p* values (*t*-test). The lines were fitted by the least squares method to a linear equation. (**b**) The growth rate in liquid media of strains expressing α-synuclein was plotted against the α-synuclein construct copy number. The strains were grown in HL-5 medium in shaken cultures at 21 °C and the generation times (doubling time during exponential growth) were measured. Experiments were performed in duplicate in each of two separate experiments. Neither the α-synuclein WT nor the mutant forms caused adverse effects on growth in liquid. Error bars represent standard errors of the mean. The regression was not significant as indicated by the p value. The line was fitted by the least squares method to a linear equation.

The *Dictyostelium* strains expressing the wild type α-synuclein, point mutant and truncated forms were grown on a lawn of *E. coli* B2 and the plaque expansion rate was measured. As shown in Figure 5a all three classes of α-synuclein-expressing strains showed slower growth compared to the parental strain, AX2. In the case of the wild type α-synuclein the reduction in growth rate ranged from 20% to 60% and was correlated tightly with the copy number. The mutant forms of the protein caused a dramatic 40–80% reduction in plaque expansion rate that also correlated with copy number. In contrast to their slow growth on bacterial lawns, the WT and mutant α-synuclein-expressing strains displayed wild type generation times in liquid (Figure 5b). This is in contrast to the known effects of mitochondrial dysfunction which impairs growth both on bacterial lawns and in liquid medium. The difference between the growth phenotypes in nutrient broth and on bacterial lawns could be due to differences in the endocytic pathways used for uptake of nutrients, phagocytosis for bacteria and macropinocytosis for liquid medium.

### 3.6. α-Synuclein Causes an Impairment of Phagocytosis, Which Results in Reduced Growth Rates on Bacterial Lawns

**Figure 6:**
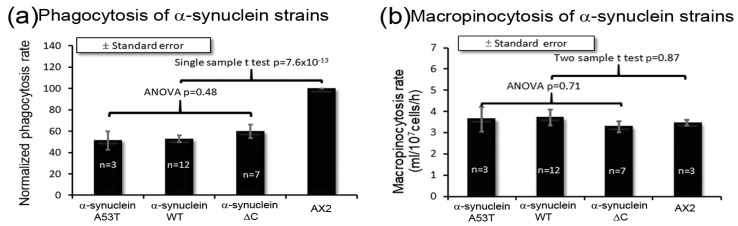
α-Synuclein affects phagocytosis but not macropinocytosis. (**a**) *Dictyostelium* amoebae were fed *E. coli* expressing Ds-Red fluorescent protein and duplicate fluorescent measurements were taken immediately after the addition of bacteria and after 30 min of incubation at 21 °C on a shaker. The uptake rates were normalized to the uptake rate for AX2. The indicated number of strains (*n*) expressing each mutant α-synuclein form all showed a similar, significantly reduced rate of phagocytosis compared to the parental strain AX2, but there were no statistical differences among them (ANOVA with pairwise comparisons using the Least Squares Difference test). In a single sample *t*-test, the normalized phagocytosis rates for strains expressing α-synuclein forms were significantly lower than 100% (the parental strain, AX2). Each strain was assayed in at least three independent experiments. (**b**) *Dictyostelium* amoebae were fed HL-5 medium containing FITC-dextran and duplicate fluorescent measurements were taken immediately after the addition of FITC-dextran and after 70 min of incubation at 21 °C on a shaker. Compared to the parental strain AX2, macropinocytosis was unaffected in all the α-synuclein-expressing strains. Cell lines were measured in at least three independent experiments and error bars represent standard errors of the mean. The difference from AX2 was not significant at indicated *p* value (two sample *t*-test) and the differences among all α-synuclein strains were not significant (ANOVA and pairwise comparisons using the Least Squares Difference test).

When feeding on bacterial lawns, *Dictyostelium* take up nutrients through phagocytosis and during growth in liquid medium, they ingest extracellular nutrients by macropinocytosis. Figure 6a shows that the phagocytosis rates were reduced due to expression of mutant forms and the WT α-synuclein, but macropinocytosis was not affected (Figure 6b). This accords with growth data and suggests that α-synuclein impairs growth on bacterial lawns because it interferes with phagocytosis. This contrasts with the phenotypic outcomes of mitochondrial dysfunction which have been reported not to affect either phagocytosis or macropinocytosis [36].

### 3.7. AMPK-Dependent and AMPK-Independent Pathways

Some of the phenotypes displayed by the α-synuclein strains mimic mitochondrial disease phenotypes including defective slug phototaxis and thermotaxis, altered fruiting body morphology with shorter thicker stalks and slow rates of plaque expansion on bacterial lawns, but others did not, in particular decreased phagocytosis and the absence of any effect on growth rates in liquid medium. In *Dictyostelium* the characteristic mitochondrial disease phenotypes are caused by chronic activation of the key sensor of cellular energy status in eukaryotes, AMPK. Expression of a constitutively active form of AMPK in *Dictyostelium* mimicked the mitochondrial defective phenotypes and knock down of AMPK in mitochondrial diseased cells rescued the defective phenotypes [36].

**Figure 7:**
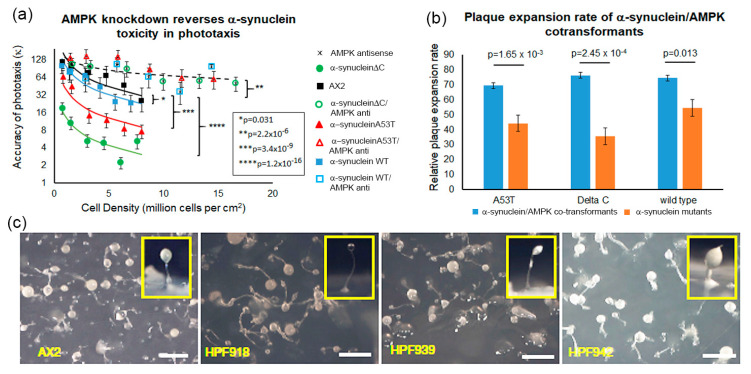
Ability of AMPK knockdown to rescue phenotypic defects caused by expression of wild type and mutant forms of α-synuclein. (**a**) Quantitative phototaxis. The parental strain AX2, α-synuclein WT HPF885 (38) and α-synuclein WT/antisense AMPK HPF918 (211, 28), α-synuclein A53T HPF902 (27) and α-synuclein A53T/antisense AMPK HPF940 (272, 153), and α-synucleinΔC HPF905 (12) and α-synucleinΔC/antisense AMPK HPF942 (215, 142) were statistically analysed and the accuracy of phototaxis (κ) was plotted against their cell densities. When compared to AX2, all the strains showed a high accuracy of phototaxis which suggests that the down regulation of AMPK rescues the phototaxis defect observed in α-synuclein-expressing strains. The multiple log–log regressions were done using the “Backwards” method to remove insignificant variables at a significance cut-off of 0.01. The resulting p values (indicated by asterisks and shown in the box inset) indicate the significance of the indicated differences (indicated by curly brackets) between the fitted regressions in the final model. Lines show the final fitted regression model and the error bars represent 90% confidence intervals for the individual data points. (**b**) Knocking down expression of AMPK partially rescues the impaired growth on bacterial lawns caused by WT or mutant α-synuclein. The plaque expansion rate of the strains expressing any of the three α-synuclein proteins were greatly reduced compared to the parental strain AX2. The cotransformants expressing WT or mutant α-synuclein and with reduced AMPK displayed partially rescued growth rates better than the single α-synuclein strains but still not as high as the parental strain AX2. All strains were grown on *E. coli* B2 lawns on SM agar and the plaque expansion was measured over a one hundred hour period. Each cell line was measured in triplicate in three independent experiments. The differences were significant at indicated *p* values (*t*-test) and error bars represent standard errors of the mean. (**c**) Knocking down AMPK does not rescue the impaired morphology caused by α-synuclein with a deletion of the C-terminus. The parental strain AX2, the WT-α-synuclein/antisense-AMPK strain HPF918, the A53T-α-synuclein/antisense-AMPK strain HPF939 and the α-synucleinΔC/antisense-AMPK strain HPF942 are shown. The WT-α-synuclein/antisense-AMPK and A53T-α-synuclein/antisense-AMPK resemble the parental strain AX2, while the α-synucleinΔC/antisense AMPK results in fruiting bodies with shorter, thicker stalks when compared to AX2. This phenotype is similar to the α-synucleinΔC strain suggesting that antisense inhibition of AMPK does not rescue the defective morphogenesis phenotype. The scale bar shows 1 mm.

To determine if increased AMPK activity mediates any of the aberrant defective phenotypes caused by α-synuclein, cotransformants were created in which AMPK expression was antisense-inhibited and one of each of the three forms of α-synuclein were also expressed. The copy numbers of the α-synuclein-expressing constructs and the AMPK antisense-inhibition constructs were determined by qPCR. The cotransformants were analysed phenotypically for their accuracy of phototaxis, fruiting body morphology, and growth on bacterial lawns. Antisense inhibition of AMPK expression completely rescued the phototaxis defect caused by all three forms of α-synuclein, with accuracies of phototaxis (κ) within the parental AX2 range (Figure 7a). Cotransformants were also analysed for their growth on bacterial lawns. Knockdown of AMPK expression in these strains caused a partial rescue of the growth defect on bacterial lawns (Figure 7b). This suggests that the plaque expansion defect caused by α-synuclein expression in *Dictyostelium* is partly caused by an AMPK-dependent mechanism and partly by an AMPK-independent mechanism. The latter could be a result of impaired phagocytosis, since phagocytosis was previously shown to be impervious to AMPK signalling [36].

In support of the hypothesis that the cytotoxicity of α-synuclein is mediated at least in part by AMPK-independent mechanisms, the fruiting body morphologies (Figure 7c) of the cotransformants resembled those of the corresponding α-synuclein-expressing transformants in which AMPK expression had not been knocked down (Figure 7c). Therefore, the fruiting body defect is produced via an AMPK-independent pathway.

### 3.8. Seahorse Respirometry

**Figure 8:**
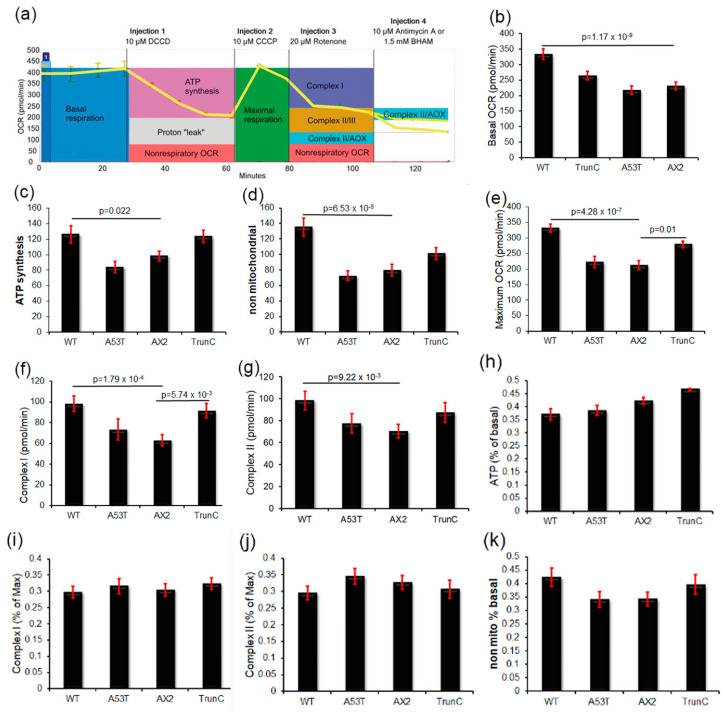
Mitochondrial respiration is not impaired in strains expressing α-synuclein. Panel (**a**): Example of a Seahorse experiment showing the raw OCR values plotted against time. Various pharmacological agents were added sequentially as indicated at the top of the panel DCCD (dicyclohexylcarbodimide, in all wells), CCCP (carbonyl cyanide m-chlorophenyl hydrazone, in all wells), Rotenone (in all wells) and either Antimycin A (in half of the wells) or BHAM (benzohydroxamic acid, in the other half of the wells). Coloured boxes illustrate how each component was measured [45]. Total Complex II activity was determined as the sum of the effects of Antimycin A and BHAM. Panels (**b**–**g**): Horizontal bars with *p* values indicate the pairwise comparisons that were statistically significant (at *p* ≤ 0.05). All other pairwise differences were not statistically significant. The parental strain AX2 and strains expressing different forms of α-synuclein (full length α-synuclein (wt) *n* = 7, α-synuclein with a point mutation at amino acid 53 (A53T) *n* = 2 and α-synuclein with a truncation of the C terminal 20 amino acids (TrunC) *n* = 8) were analysed by Seahorse Respirometry. Each strain was assayed over four replicates in an average of 3–6 independent experiments. Significant differences from the parental strain AX2 are indicated in the *p* values (*t*-test). Error bars are standard errors of the mean. Basal OCR (**b**), OCR dedicated to ATP synthesis (**c**), nonmitochondrial OCR (**d**), Maximum OCR Complex I (**f**), and Complex II (**g**). Expression of wild type α-synuclein (WT) resulted in increased mitochondrial respiration and an increase in oxygen consumption by nonmitochondrial processes, while expression of α-synuclein with a point mutation (A53T) had no significant effect on respiration and the C-terminal truncation mutant (TrunC) increased the maximum respiratory capacity of the cells. Panels (**h**–**j**): Each of the parameters was plotted as a proportion of Basal or Maximum OCR and found to be unchanged, which shows that all respiratory complexes were functionally normal and so made the normal relative contribution to respiration. Shown are the OCR attributable to ATP synthesis as a % of Basal OCR (**h**), the relative contribution of Complex I (%) to the Max OCR (**i**), the relative contribution of Complex II (%) to the Max OCR (**j**) and non-mitochondrial OCR as a % of Basal OCR (**k**).

The foregoing data suggest that expression of α-synuclein in *Dictyostelium* results in the activation of AMPK perhaps through an impairment of mitochondrial function. To measure mitochondrial function directly in α-synuclein-expressing strains in real time, the Seahorse Extracellular Flux Analyser was used in combination with a series of drugs added in a sequential order. This enabled various parameters of mitochondrial respiration to be measured using the Oxygen Consumption Rate (OCR) as a readout of mitochondrial respiration. These include the rates of oxygen consumption attributable to basal respiration (Basal OCR) and ATP synthesis (ATP) as well as the maximum respiratory capacity of the cells (Max OCR), the “proton leak” (use of the mitochondrial proton gradient by processes other than ATP synthesis) and oxygen consumption by other non-mitochondrial enzymes (non-mitochondrial OCR). Figure 8a shows an example of a Seahorse respirometry experiment and highlights how each measure was calculated. The α-synuclein-expressing strains showed a trend towards increased respiratory activity in some (A53Tα-synuclein) or all (WT and truncated forms of α-synuclein), but only in the case of strains expressing the wild type protein did this reach statistical significance for every component of respiration (Figure 8b–g). Elevation of the maximum respiratory capacity and its major component, uncoupled Complex I activity, also reached statistical significance in strains expressing the truncated form of the protein. There was no significant inhibition of the relative contribution (as a percentage of basal or maximum OCR) of any component of mitochondrial respiratory function by either the wild type or mutant forms of α-synuclein. These results are reminiscent of those observed in several different cellular PD models—knockdown of the PD-associated protein DJ-1 in *Dictyostelium* cells [55], ectopic treatment of cultured human neuroblastoma cells with oligomeric α-synuclein fibrils [56] in lymphoblasts isolated from idiopathic PD patients [57]. As in those cases, the results show that activities of the mitochondrial respiratory complexes are elevated or unchanged, not decreased, and that the complexes remain functionally normal (making normal relative contributions to total respiratory activity). For example, if Complex I had been specifically inhibited by α-synuclein it would have made a proportionately smaller contribution to the total uncoupled, maximum OCR.

## 4. Discussion

Alpha-synuclein is a small soluble protein which predominantly localises to specific membranes in neurons such as presynaptic vesicles and the mitochondria. It has many postulated roles in the neurons such as in synaptic transmission, intracellular protein trafficking and axonal transport. Binding to membranes is facilitated mainly via its amphipathic α-helical domain in the N terminus [58].

Here we expressed α-synuclein and two mutant α-synuclein (a point mutation A53T and a C-terminal truncation) proteins in *Dictyostelium*. The wild type and A53T α-synuclein concentrated in the cell cortex, in agreement with its known association with biological membranes. This is also in agreement with studies in yeast in which α-synuclein wild type and A53T were expressed and showed preferential localisation to the cell cortex with additional localisation in the cytoplasm [59,60]. In contrast, the C-terminally truncated α-synuclein protein did not localise at the cell cortex but was found throughout the cytoplasm. Importantly, expression of GFP tagged with only the 20 most C-terminal amino acids of α-synuclein in *Dictyostelium* resulted in concentration of this peptide to the cell cortex suggesting that these amino acids are sufficient for localisation of α-synuclein to the cell cortex. The C-terminal 20 residues have been expressed in *E. coli* and as in *Dictyostelium* this portion of the protein was important for localisation of the protein, but in *E. coli* this location was the periplasm [61]. The N-terminus was not required for this translocation to the periplasm and it was unlikely to involve phospholipid binding. However, the relevance of this finding to eukaryotic cells like *Dictyostelium* is unclear, since eukaryotes do not have a subcellular compartment equivalent to the periplasm which lies between the cytoplasmic and outer membranes of Gram-negative bacteria.

The C-terminus of α-synuclein is also a major site for post translational modifications and is important for localisation of the protein to the nucleus, protein–protein interactions, and interactions with metal cations like Ca^2+^ [62,63,64,65,66,67]. Deletion of the C terminus has also been associated with an increased propensity of the protein to aggregate. In *Dictyostelium*, the involvement of the C-terminus suggests that the cortical localization of α-synuclein is unlikely to be due to phospholipid binding, but is perhaps mediated through an interaction between the C-terminus and a protein which is itself localised to the cell cortex.

Expression of all three forms of α-synuclein resulted in impaired growth on bacterial lawns but had no effect on axenic growth. Expression of α-synuclein in yeast also caused growth defects [60] but no effect on growth was observed in *E. coli* [61]. This suggests that the cytotoxic effects on cell growth are mediated by mechanisms specific to eukaryotic cells. What eukaryote-specific pathways might be involved? One clue comes from the differences in the effect of α-synuclein on growth on different nutrient sources in the experiments reported here. Our results showed that these are likely due to the different modes of endocytosis employed during growth on bacteria and in liquid medium. *Dictyostelium* lives in forest soil and ingests its microbial food source by phagocytosis while most laboratory strains are also able to grow axenically using macropinocytosis. The two processes of endocytosis have many components and proteins in common, but there are also differences between them. Both involve actin-dependent, clathrin-independent mechanisms. In addition to actin cytoskeletal rearrangements, phagocytosis also relies upon adhesion factors and receptors for recognizing bacterial prey. Mutations to several of these proteins such as SadA [68], SibA and SibC (similar to β-integrins) [69] and PhtgA [70] result in defects in phagocytosis but have no effect on macropinocytosis. Several other proteins have been shown to play a role in one endocytic pathway and not the other, including signalling molecules, RacC [71], Rap1 [72], actin binding and motor proteins, profilin [73], and myosin VII [74], kinases PI3K anda lysosomal integral membrane protein (LimpA) [73]. Alpha-synuclein may mediate its effect on phagocytosis via interactions with protein(s) specific to this pathway. In mammalian cells phagocytosis has been shown to be reduced in pluripotent stem cell-derived macrophages with a triplication of the α-synuclein gene [75] and also when overexpressed in mouse macrophage [76] and in BV2 microglial cells [77]. On the other hand, there are no reports that macropinocytosis is impaired by α-synuclein in humans or other mammals, although macropinocytosis has been reported to mediate the uptake of exogeneous α-synuclein aggregates [78] or α-synuclein containing exosomes [79] from the extracellular milieu. This may contribute to the spread of α-synuclein toxicity in the brain. We have not tested whether macropinocytosis similarly mediates uptake of toxic forms of α-synuclein in *Dictyostelium*, but the cytotoxic effects of α-synuclein on phagocytosis (and not macropinocytosis) suggest that *Dictyostelium* can provide a useful model for α-synuclein cytotoxicity.

In *Dictyostelium*, mitochondrial dysfunction results in a clear set of phenotypes. As well as inhibiting growth, it causes defects in phototaxis and abnormal culmination to produce fruiting bodies with short, thick stalks. Here we found that expression of both A53T and truncated mutant forms of α-synuclein resulted in reduced accuracy of phototaxis, while an altered fruiting body morphology was observed only in the α-synucleinΔC strains. Even allowing for the different levels of toxicity of the three forms of α-synuclein, our results showed that the protein causes some phenotypes which were consistent with mitochondrial dysfunction (such as impaired slug phototaxis and impaired growth on bacterial lawns) and others which were not (including impaired phagocytosis and normal growth in liquid medium). These different patterns of abnormal phenotypes suggest that α-synuclein cytotoxicity in these cells is not mediated by mitochondrial dysfunction.

To pursue this question further, we took advantage of the fact that the characteristic phenotypic outcomes of mitochondrial dysfunction in *Dictyostelium* cells result from chronically hyperactive AMPK [36,80]. Knockdown of AMPK expression in mitochondrially diseased strains rescued all of the abnormal phenotypes, while overexpression of AMPK faithfully phenocopied mitochondrial disease. In a similar vein in this work, we examined whether AMPK knockdown could reverse the cytotoxic effects of α-synuclein. While AMPK antisense inhibition completely rescued the phototaxis defect, it only partially rescued the defect in plaque expansion on bacterial lawns and had no influence on the fruiting body abnormalities in the α-synucleinΔC strains (or on the phagocytosis defects). This implies that in *Dictyostelium*, some α-synuclein cytotoxic effects are AMPK-dependent and others result from AMPK-independent pathways.

Despite the overall pattern of α-synuclein cytotoxicity phenotypes being discordant with mitochondrial dysfunction, it remained possible that the AMPK-dependent subset of these phenotypes resulted from AMPK hyperactivity elicited by mitochondrial dysfunction and energy stress. However, direct respirometric measurement of mitochondrial function in live cells revealed that none of the three tested forms of α-synuclein caused significant impairment of mitochondrial respiratory function. In fact, there was a broad trend towards elevated respiratory activity in the α-synuclein-expressing strains. This reached statistical significance in all components of respiration in cells expressing wild type α-synuclein and in the maximum respiratory capacity and Complex I activity in cells expressing the C-terminal truncation mutant. In addition, all complexes in the respiratory chain were functioning normally, as revealed by the fact that their individual fractional contributions to overall respiratory function were unchanged. Similar increases in mitochondrial function have been reported recently in several other cell-based Parkinson’s Disease models. Knockdown of the *Dictyostelium* homologue of DJ-1, whose loss of function in humans causes PD, resulted in increased mitochondrial respiration and its overexpression resulted in decreased respiration [55]. Neuroblastoma cells exposed to cytotoxic α-synuclein fibrils, but not non-toxic monomers, exhibited increased respiratory function [56]. Furthermore, mitochondrial activity was similarly elevated in lymphoblasts from idiopathic PD patients compared to controls [57]. Our results here are in agreement with those studies and again suggest that *Dictyostelium* provides a valuable model for α-synuclein cytotoxicity. One explanation of the lack of any significant effect on mitochondrial function of the A53T mutant form of α-synuclein is the small sample of independent transformants used—only two of these strains were tested in the Seahorse respirometer. Alternatively, the A53T mutation could cause a loss of the protein’s ability to stimulate mitochondrial respiration in cells.

The increase rather than impairment of mitochondrial function observed in the *Dictyostelium* cells expressing α-synuclein suggests that AMPK is not activated by α-synuclein via a defect in energy production, but by one of the other diverse cellular stressors to which AMPK responds such as glucose deprivation [81,82], redox stress [83] oxidative stress [84] and nutrient stress (low amino acid supply) [85,86].

## 5. Conclusions

Our results show that the *Dictyostelium* model can provide a useful system in which to study the normal roles of α-synuclein and the cytopathological pathways leading to synucleinopathies like Parkinson’s disease. We expressed wild type α-synuclein and two PD-associated mutations of α-synuclein in the eukaryotic model *Dictyostelium discoideum*. Alpha-synuclein has been reported to localise to multiple compartments within cells. In *Dictyostelium* we showed here that the C-terminus of α-synuclein is both necessary and sufficient for localisation of the protein to the cell cortex. Expression of wild type or mutant α-synuclein proteins was toxic to the cells resulting in a growth defect on solid media that was in part caused by a defect in phagocytosis. The phagocytosis defect is in agreement with mammalian cell models of α-synuclein toxicity. *Dictyostelium* can also employ another mode of endocytosis called macropinocytosis to ingest nutrients. We demonstrate here that α-synuclein selectively disrupts phagocytosis and not macropinocytosis. Mitochondrial respiration was elevated in *Dictyostelium* strains expressing α-synuclein and suggests that the dogma of impaired mitochondrial function in Parkinson’s disease may need to be further investigated. Instead, our results support the emerging view that α-synuclein may exert its cytotoxic effects primarily on endocytic and vesicle trafficking pathways [87], with the reported mitochondrial damage in postmortem PD brains being a secondary, subsequent consequence of the disease process [57,82].

## Figures and Tables

**Figure A1:**
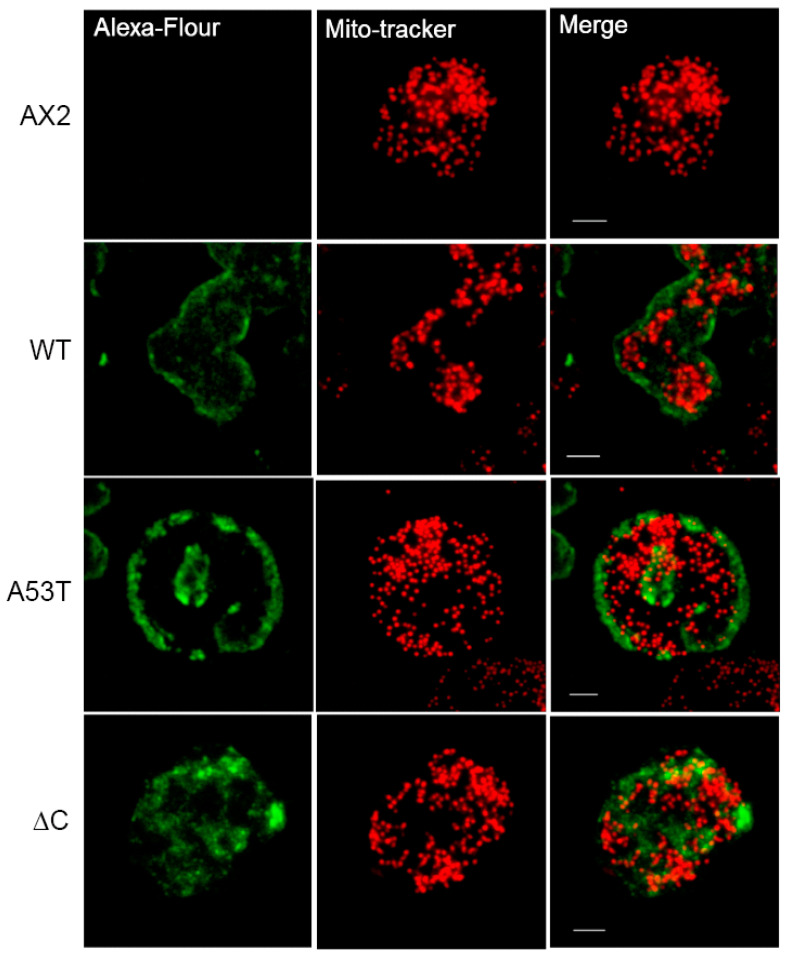
α-synuclein does not colocalise with mitochondria.

## Appendix A. α-Synuclein Does Not Colocalise with Mitochondria

Confocal microscopy detecting α-synuclein and mitochondria. The first column shows α-synuclein using a secondary antibody conjugated to Alexa Fluor 488, the second column shows mitochondria (Mitotracker Red) and the final column is a merge of the first two columns. Both WT and point mutant forms (A53T) of α-synuclein accumulate close to the cell cortex and the truncated α-synuclein (ΔC) was present granulated throughout the cytoplasm as in Figure 2. The oblong ring-shaped region of A53T-α-synuclein near the centre of the cell (column 1, row 3) is located in the cortical region of a ruffle at the dorsal surface of the cell. None of the three forms of α-synuclein showed any colocalization with mitochondria. Scale bar = 3 µm.
